# Summer UTCI variability in Poland in the twenty-first century

**DOI:** 10.1007/s00484-020-01965-2

**Published:** 2020-07-17

**Authors:** Agnieszka Krzyżewska, Sylwester Wereski, Mateusz Dobek

**Affiliations:** Department of Hydrology and Climatology, Faculty of Earth Sciences and Spatial Management, University of Maria Curie Skłodowska in Lublin, Al. Kraśnicka 2CD, Lublin, Poland

**Keywords:** UTCI, Poland, Bioclimatic regions, Summer, Heat wave 2015

## Abstract

The paper analyses the temporal and spatial variability of the Universal Thermal Climate Index (UTCI) in Poland in summer. Summer is the season with the highest intensity of tourism traffic that is why it is important to determine biometeorological conditions, especially in popular tourist destinations such as coastal, mountain and urban areas, in the times of climate changes. The analysis was based on data from 18 stations of IMGW-PIB (Institute of Meteorology and Water Management—National Research Institute), distributed evenly in the territory of the country, and representing all eight bioclimatic regions. The data include air temperature, relative humidity, wind velocity and cloudiness at 12 UTC from summer months: June, July and August from the years 2001–2018. *Thermoneutral zone* was the most frequently occurring UTCI class in Poland*.* It was recorded during 56–75% of summer days (with the exception of mountain stations, where it occurred on 30–35% of days). *Moderate heat stress* is the second most frequently occurring category with a frequency from 18 to 29% with the exception of mountain and coastal areas. *Extreme* and *very strong cold stress* occurred particularly in high mountain stations, and was sporadically observed at the coast of the Baltic Sea; however, the occurrence of such conditions decreases, which if favourable for beach tourism. No cases of *extreme heat stress* were recorded in any of the stations. The most unfavourable bioclimatic conditions were characteristic of the Upland Region (IV), represented by Kraków and Sandomierz, where *very strong heat stress* occurred with a 10% frequency. This is a limitation for urban tourism in those regions. The highest UTCI values were recorded in Kraków on 17 July 2007 and 29 July 2005. The highest number of cases with *strong* and *very strong heat stress* was recorded in 2015 as a consequence of the heat wave observed in Poland in the first half of August. In the majority of the analysed stations, in the second half of the analysed period (2010–2018), an increase in the number of days with *strong* and *very strong heat stress* was observed in comparison with the first half of period (2001–2009). The highest frequency of such days was observed in July. Based on the data, there are 4 potential periods of occurrence of such days, with two most intense being 26. July–13 August and 14–22 July.

## Introduction

In Poland, summer is the season with the highest intensity of tourist traffic, with a maximum in July and August. The seasonal distribution of tourist traffic is considerably affected by weather and climatic conditions, which are one of the most important factors for outdoor tourism (Matzarakis 2006). Despite the possibility and wide variety of offers of foreign travels, still about 80% of Polish people choose domestic destinations (Łysoń 2019). Two most popular destinations in the country are (1) the coastal region at the Baltic Sea in the north (Koźmiński and Michalska 2016) and (2) mountain areas in the south, in the Carpathians and Sudetes. The number of accommodation places used by tourists in those regions constitutes almost half of all accommodation places used by tourists throughout Poland in 2018 (Łysoń 2019). However, those two areas in summer are colder than the rest of the country (Tomczyk and Owczarek 2020).

Due to contemporary climate change, some touristic destinations can have more or less favourable conditions for different types of tourism (WMO, UNEP 2008). Such changes can be observed especially in recent decade, which was announced to be the warmest decade since pre-industrial times and 2019 was the second (after 2016) warmest year (public.wmo.int). For example, Mediterranean area can be too hot and dry and Scandinavian countries may have more favourable conditions for outdoor tourism (WMO, UNEP 2008). During summer, some regions will experience more climate extremes related to high temperature, for example heat waves, which will occur increasingly frequently, and will be longer and more intensive (IPCC 2014). Such unfavourable meteorological conditions lead to a higher number of deaths (Kuchcik 2017). Moreover, this type of phenomena has a very negative effect on human health and life, the environment, tourism and economy (Krzyżewska 2015). However, to analyse the influence of weather conditions on human health, single meteorological parameters are not enough, but a complex index is needed, which comprises all relevant meteorological parameters like air temperature, air humidity, wind speed and radiation in assessing heat load (Matzarakis and Nastos 2011). The most comprehensive description of the effect of meteorological elements on man is offered by the analysis of the body heat balance, subject to numerous studies. Streams of heat exchange between the human body and the natural environment, obtained by means of modelling, are used in the so-called complex biometeorological indices. However, they do not fully reflect physiological responses occurring in the human organism in given meteorological conditions. An international research team was appointed to solve this problem. Based on a long-term research, the team developed the Universal Thermal Climate Index (UTCI) (Jendritzky et al. 2009; Jendritzky et al. 2012). UTCI values were ascribed a scale of heat stress of the organism that is devoid of subjectivity in the perception of the effect of the atmospheric environment on the human organism. This allows for among others comparison of results of studies conducted in different climatic zones (Błażejczyk et al. 2010).

It was proven that UTCI better reflects temporal thermal variability, especially with hot conditions, than indices like Heat Index, Wet-bulb globe temperature, humidex and slightly better from apparent temperature and effective temperature, and it corresponds the best with other indexes based on human health balance models, like Physiological Equivalent Temperature, perceived temperature and standard effective temperature (Blazejczyk et al. 2012; Zare et al. 2018). Also, because UTCI is very sensitive to changes in ambient stimuli (like human body), it better represents different climate, weather and location conditions than other indices, which are reasonable only under specific meteorological situations (Blazejczyk et al. 2012; Mölders 2019). Also, evaluated clothing model is implemented in UTCI which considers human behaviour (Havenith et al. 2012).

UTCI is the most advanced biometeorological index, which is confirmed by the studies of Jendritzky et al. (2012) and Katavoutas and Founda (2019). It is widely applied in multiple practical approaches, like short- and long-term influence of the atmospheric environment on human body, in medical studies, in many aspects of tourism research or in urban bioclimate (Staiger et al. 2019). It is useful for example in Public Weather Service, Public Health Service, Precautionary Planning, or Climate Impact Research (Błazejczyk et al. 2013). Examples of papers with a regional range employing UTCI include studies for selected regions of Poland (Błażejczyk and Kunert 2010; Chabior 2011; Milewski 2013; Koźmiński and Michalska 2019). Chabior (2011) and Koźmiński and Michalska (2019) applied UTCI to analysis of bioclimate conditions of the Baltic coast—one of the main tourist destinations in Poland, especially for recreational tourism in summer seasons. Chabior (2011) suggested using UTCI to determine thermal sensations based on scale by Baranowska (Baranowska and Gabryl 1981). Koźmiński and Michalska (2019) based on frequency of occurrence of unfavourable days (strong cold stress and very strong cold stress) draw the zones of different heat load in the Baltic region. For mountain regions, UTCI was used by Błażejczyk and Kunert (2010) and Miszuk et al. (2016). In both papers, the authors focused on high variability of bioclimatic conditions in areas of varied relief.

For the entire country, UTCI was used by Kuchcik et al. (2013), Błażejczyk et al. (2015) and Kuchcik (2017). One of the main problems in those publications was the influence of bioclimate conditions on health of Polish Citizens, particularly during periods with strong cold and heat stress.

The UTCI index has been frequently used in research on heat stress in urbanised areas. The highest number of papers of the type concerns the area of Warsaw (Lindner 2011; Błażejczyk et al. 2014; Rozbicka and Rozbicki 2018). Błażejczyk et al. (2014) analysed biothermal conditions of Warsaw in the contex of urban heat island. They also drew attention to the role of building density and the presence of biologically active areas in the city in shaping biothermal conditions, especially in the summer seasons. In studies on other cities, UTCI was applied by Błażejczyk et al. (2010) in Łódź, Dobek et al. (2013), Dobek and Krzyżewska (2015) and Krzyżewska et al. (2016) in Lublin, Nidzgorska-Lencewicz (2015) in Gdańsk or Bryś and Ojrzyńska (2016) in Wrocław. Dobek et al. (2013) for Lublin, as well as Milewski (2013) for Kłodzko Land, presented spatial variability of bioclimatic conditions under different weather scenarios, based on relief, spatial management and soil moisture. Krzyżewska et al. (2016) analysed biothermal conditions in Lublin during the extreme heatwave event of August 2015. In the publication by Nidzgorska-Lencewicz (2015) besides analysis of biothermal conditions in Tricity (Gdańsk, Gdynia and Sopot metropolitan area), there was an assessment of air quality based on Common Air Quality Index (CAQI).

The dependency of UTCI on the atmospheric circulation was analysed by Nowosad et al. (2013) in Lublin and Lesko, Bartoszek et al. (2017) in Lublin, Rozbicka and Rozbicki (2018) in Warsaw, Owczarek et al. (2019) or Tomczyk and Owczarek (2020) in Poland. In the last paper, authors focused only on *strong* and *very strong heat stress* cases.

However, none of the abovementioned papers covers the complex and detailed analysis of all classes of UTCI variability in summer, both spatial, including the whole Poland, and temporal, including most recent changes in the twenty-first century. The objective of this paper is (1) spatial and (2) temporal analysis of biometeorological conditions in bioclimatic regions of Poland during the summer season, featuring the highest intensity of tourist traffic. The analysis puts special focus on favourable and unfavourable biometeorological conditions for different types of outdoor tourism (coastal tourism, urban tourism, active tourism) in different bioclimatic regions during the whole summer, but also in particular summer months. Additionally, the periods of increased frequency of days with *strong* and *very strong heat stress* are distinguished, which affects various types of tourism differently.

## Material and methods

### Study area

Poland is located in a transitional area between two major climate zones according to Köppen division: Cfb (warm temperature, fully humid, warm summer) in the western part and Dfb (snow, fully humid, warm summer) in the eastern part (Kottek et al. 2006). Climate and bioclimate are shaped by different factors, like solar radiation, atmospheric circulation (with main air masses Polar Maritime and Polar Continental), geographical location and local features (Błażejczyk 2006).

**Fig. 1:**
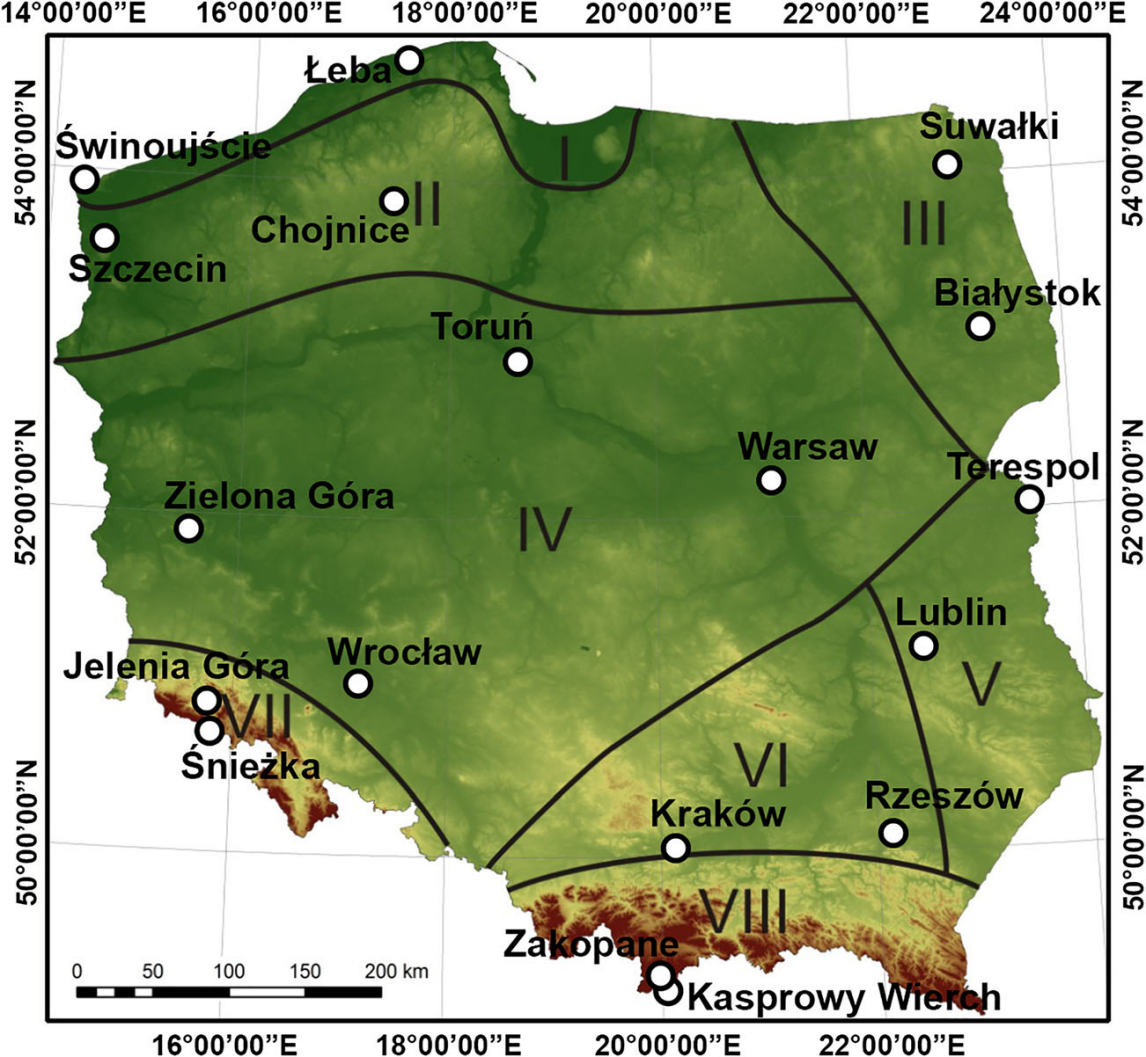
Distribution of selected meteorological stations on the background of bioclimatic regions of Poland

Poland (Fig. 1) is divided into eight bioclimatic regions (Błażejczyk and Kunert 2011). The northernmost region is the Coastal Region (I), represented by two stations: Świnoujście, near the west border of Poland, and Łeba—the northernmost station considered in the analysis. The region is under a strong influence of the Baltic Sea, and is characterised by stronger stimuli of thermal environment. The second bioclimatic region—the Lakeland Region (II)—is characterised by slightly lower stimuli of thermal environment (Kozłowska-Szczęsna et al. 1997). It includes two stations considered in the analysis, namely Szczecin (in the west) and Chojnice (the middle part). In the north-east of Poland, the North-Eastern Region (III) is located, with stations in Suwałki and Białystok. Besides mountain areas, this region is considered the coldest. The Central Region (IV) covers the largest, central part of Poland, and is characterised by weak stimuli of thermal environment (Kozłowska-Szczęsna et al. 1997). Due to its size, the analysis considered 4 stations, namely Zielona Góra and Wrocław in the west of the region, Toruń in the north of the region, and Warsaw a bit further to the east. In the South-Eastern Region (V), the analysis covered Lublin and Terespol, and in the Upland Region (VI)—Kraków and Rzeszów. They are the warmest regions in the country (Kozłowska-Szczęsna et al. 1997).

Two mountain regions are located furthest to the south, namely the Sudetic Region (VII) in the west and Carpathian Region (VIII) in the east. The Sudetic Region (VII) is represented by a mountain station on Śnieżka (1603 m a.s.l.), and Jelenia Góra located lower (342 m a.s.l.). The Carpathian Region (VIII) is represented by Kasprowy Wierch (1991 m a.s.l., mountain station) and Zakopane (857 m a.s.l). Both regions are distinguished by high variability of bioclimatic conditions, and strong stimuli of thermal environment. They are also the coldest regions (Kozłowska-Szczęsna et al. 1997).

### Data

The paper is based on measurement and observation term data (air temperature, relative humidity, wind speed and cloudiness) from 18 meteorological stations of the Institute of Meteorology and Water Management—National Research Institute. The meteorological data cover summer months: June, July, and August in the period 2001–2018 at 12 UTC. The noon term of observation is the most representative time of day for human activity (Kozłowska-Szczęsna et al. 1997).

The meteorological stations were selected in a way to be complete, represent all biometeorological regions of Poland, and be distributed very evenly in the territory of the country (Fig. 1). The presentation of mean seasonal thermal conditions employed mean daily temperature. The detailed analysis of biothermal conditions that occurred during the heat wave in August 2015 covered 24-h observations.

### UTCI

UTCI is defined as “equivalent temperature for a given combination of wind speed, radiation, humidity and air temperature (…) as the air temperature of the reference environment which produces the same response index value” (Jendritzky et al. 2012).

UTCI is based on an expanded model of the human heat balance model, the so-called Fiala Model (Fiala et al. 2001; Fiala et al. 2012), considering two parameters of heat exchange regulation between the human organism and the surroundings. The first one, the so-called passive, covers heat transport inside the organism and on the body surface. The second one, called active, determines the physiological mechanisms of thermoregulation. Values of the UTCI index expressed in (°C) are obtained based on multiple calculation of the human heat balance. This provides further parameters necessary for obtaining the final result. The UTCI index can be expressed as the following function:

$$ \mathrm{UTCI}=\mathrm{f}\ \left(\mathrm{Ta},\mathrm{vp},\mathrm{va},\mathrm{dTmrt}\right) $$

where

- Ta—air temperature (°C),
- vp—water vapour pressure (hPa),
- va—wind speed at a height of 10 m above ground level (m·s^−1^),
- dTmrt—difference between mean radiation temperature and air temperature (°C).

Tmrt, called mean radiation temperature, reflects values of temperature of a thin layer of air surrounding the human body. The value is shaped by streams of short- and long-wave radiation reaching the body (COST Action 730). Details of the structure of the index, description of all its components and verification are included among others in papers by K. Błażejczyk et al. (2012), P. Bröde et al. (2012), D. Fiala et al. (2012), G. Haventih et al. (2012), B. Kampmann et al. (2012) and A. Psikuta et al. (2012). Table 1 presents classes of heat stress of the human organism according to the UTCI index.

**Table 1:**
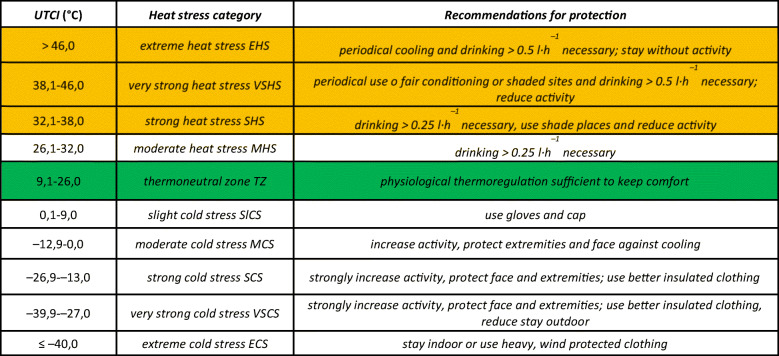
Assessment scale of UTCI (Błazejczyk et al. 2013)

The calculation of the UTCI value employed Bioklima ver. 2.6. Software 2010 (https://www.igipz.pan.pl/Bioklima-zgik.html). Because measurements of solar radiation intensity, necessary for the determination of the mean radiation temperature value, were not conducted in all stations, the calculations were based on information on cloudiness, and applied a radiation model implemented to the BioKlima software. In all cases where wind speed was equal to 0 m/s, value 0.5 m/s was adopted, and when it exceeded 20 m/s, value 20 m/s was adopted. This was done to follow the UTCI authors’ guidelines for index calculation when wind speed is from 0.5 to 17 m/s (http://www.utci.org/utci_doku.php). Novak (2013) shows that with wind speeds of 20–30 m/s the value of UTCI increases, when we should expect higher cooling effect. With further increase of wind speed, UTCI index decreases very quickly to values below – 273.15 °C (0 K). Novak suggested a solution to use a wind speed equal to 20 m/s for all values which are above 20 m/s.

## Results

### Mean summer temperature

Thermal conditions in the summer period are variable depending on the bioclimatic region and location of the station. The coldest places in summer included high-mountain stations: Kasprowy Wierch (Carpathian Region, 1991 m a.s.l.), with mean summer temperature of 9.8 °C, and Śnieżka (Sudetic Region, 1603 m a.s.l.), with mean summer temperature of 10.2 °C. Zakopane, located at a height of 857 m a.s.l., reached a mean summer temperature in the twenty-first century of 18.9 °C (Table 2).

**Table 2:**
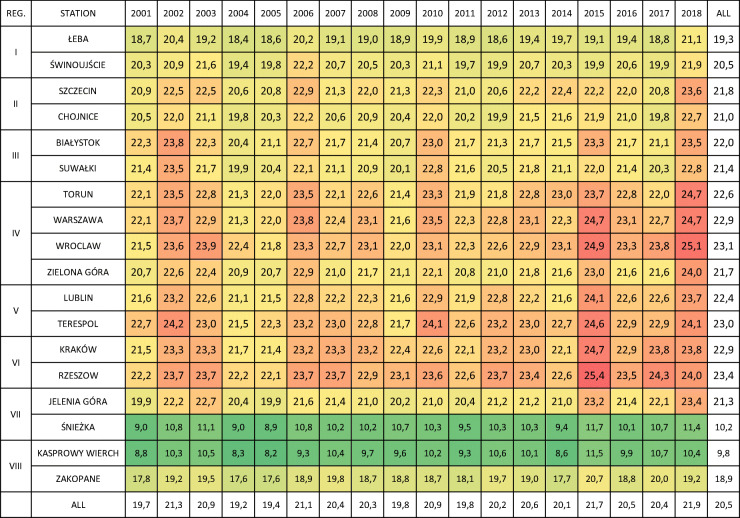
Average summer (JJA) temperature (°C) at selected Polish stations (2001–2018). Colour scale is from green (lowest values) to red (highest values)

Mean summer temperature was slightly higher in Łeba, where it reached 19.3 °C, i.e. the lowest value recorded outside mountain areas (the station is located approximately 11 m a.s.l.). In the second of the analysed stations of the Coastal Region—Świnoujście—mean summer temperature was 20.5 °C. Although the regions are colder, they are the most popular tourist destinations in summer. In the remaining regions, mean summer temperatures exceeded 21 °C, and the highest values were observed in Rzeszów (23.4 °C, Upland Region) and Wrocław (23.1 °C, Central Region).

Mean summer temperatures were characterised by relatively high variability in the twenty-first century. Particularly, hot years include 2002, 2015 and 2018, as observed especially in the Central and Upland Region; however, only in 2015 there was observed very long and severe heatwave, referred to as mega-heatwave (Krzyżewska and Dyer 2018). Other summers did not experience such extreme events—elevated average temperature does not necessarily need to correspond with heatwaves. Summer of 2003 that brought several tens of thousands deaths in Western Europe (Robine et al. 2008), similarly as summer 2010 in Eastern Europe, was not determined as particularly hot in Poland. The coldest summers included 2001, 2004 and 2005 (Table 2).

### Frequencies of occurrence of particular UTCI classes

**Fig. 2:**
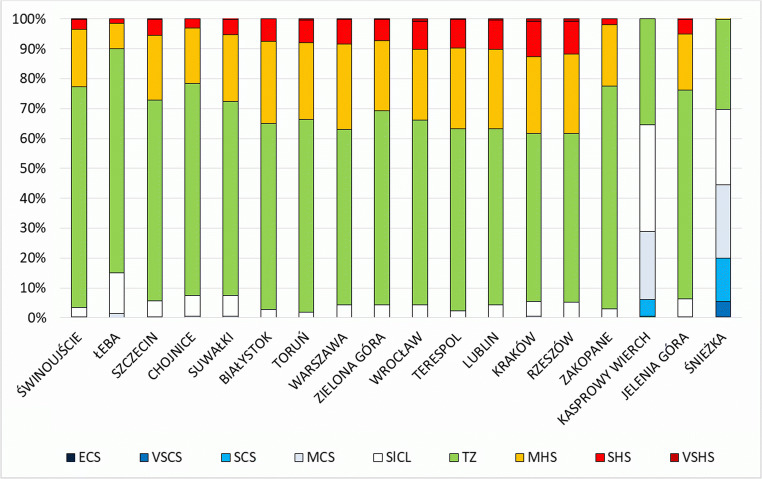
Frequency (%) of various thermal stress categories (UTCI) at 12.00 UTC in summer season (JJA) in Poland (2001–2018)

The most frequently occurring class of heat stress in Poland in the twenty-first century was *thermoneutral zone*. In lowland areas, the frequency of this class varied from 56.4 in Rzeszów and Kraków to 75.0% in Łeba. An exception were high mountain stations, where the class describing *thermoneutral* conditions accounted for only 30–35%, and cold stress conditions were prevalent (Fig. 2).

The second most frequent class of heat stress was *moderate heat stress*. Its frequency varied from 8.6 in Łeba to 28.6% in Warsaw. *Strong heat stress* usually occurred in stations of the Upland Region: 11.7% in Kraków and 10.7% in Katowice. In the remaining area, with the exception of the mountains, the frequency of the class does not exceed 10%. The lowest value occurred in Łeba, and equalled 1.4%. *Strong heat stress* occurred sporadically on a part of the analysed stations, although its frequency did not exceed 1% (0.9% of cases were recorded in each of the stations of the Upland Region, in Cracow and Rzeszów) (Fig. 2). No cases of *extreme heat stress* were recorded in any of the stations.

Because the biometeorological conditions in Poland are shaped not only by latitude, determining the inflow of solar radiation, but also by circulation conditions (Woś 1999), in summer in lowland areas, also *slight cold stress* and *mild cold stress* were observed. In lowland areas, they most frequently occurred in Łeba (13.6% and 1.3% respectively), which distinguishes the station among other stations, where the values did not exceed 7.0% and 0.6%, respectively (Fig. 2). It is worth mentioning that in addition to the fact that the station in Łeba is located furthest to the north among all stations considered in the analysis, it is also located the nearest to the sea and therefore shows lower temperature and higher wind speed than the remaining lowland stations.

Mountain areas were characterised by relatively high frequency of occurrence of classes *slight cold stress* (35.9% on Kasprowy Wierch and 25.1% on Śnieżka) and *moderate cold stress* (22.8% and 24.6%, respectively). *Strong cold stress* and *very strong cold stress* occurred more often on Śnieżka (14.5% and 5.2%, respectively) than on Kasprowy Wierch (5.5% and 0.5%, respectively). On Śnieżka, cases of *extreme cold stress* were also recorded (0.2%), caused by high wind speed values observed in the station even in summer months (Fig. 2).

### Conditions of thermal comfort (TZ, UTCI from 9.1 to 26.0 °C)

**Fig. 3:**
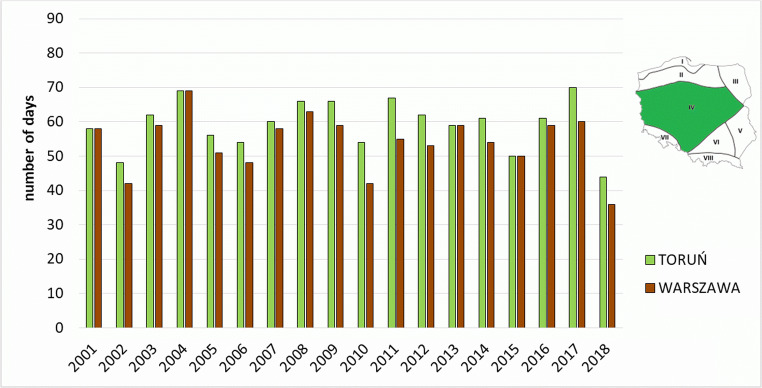
Number of days with biothermal comfort (thermoneutral zone) on the meteorological stations in Central Region (IV)

The Central Region is the largest of the analysed regions and represents biometeorological conditions typical of the majority of the country. Based on the example of stations located in its area, the variability of conditions of thermal comfort was presented. In particular years, the number of days with this category of heat stress ranged from 44 to 70 days in Toruń and from 36 to 69 days in Warsaw (Fig. 3). Such conditions are considered the most favourable for the development of tourism and recreation (Ge et al. 2017).

### Conditions of strong heat stress (SHS + VSHS, UTCI > 32 °C)

In summer months, it is considerably more important to trace the number and changes in the number of days on which the human organism may be subject to unfavourable biothermal conditions. It should be remembered, however, that the expectations concerning weather among tourists choosing passive recreation at a beach are different than among tourists practicing active forms of tourism such as hiking or cycling. In the first case, conditions of thermal comfort or slight heat stress are desirable, and in the second case, thermoneutral conditions or conditions of slight cold stress.

**Fig. 4:**
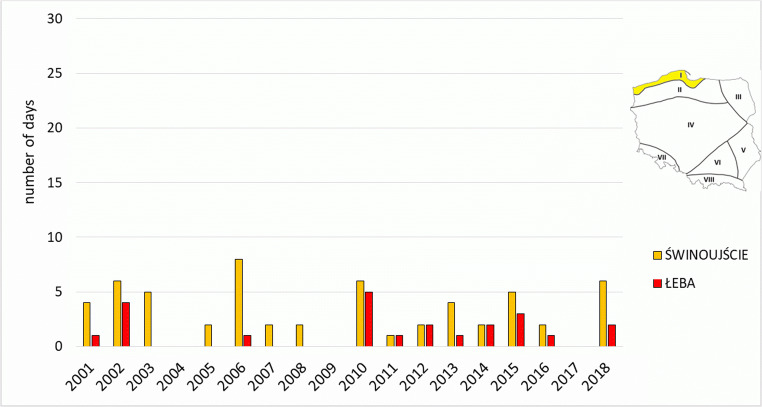
Number of days with heat stress categories (SHS + VSHS) on the meteorological stations in Coastal Region (I)

In the Coastal Region, outside mountain areas, the least cases with *strong* and *very strong heat stress* occurred. They did not occur in all years, however. In summer, this region (besides Sudetic and Carpathian) is the coldest, also windy, which is reflected in low frequency of *strong heat stress* and *very strong heat stress*. The highest number of such days was recorded in 2006 in Świnoujście (8 days) and in 2010 in Łeba (5 days). In the first half of the analysed period, they occurred considerably more seldom than in its second half, as reflected in the example of Łeba, where in the years 2001–2009 such days occurred sporadically, and since 2010 they have been occurring almost every year (Fig. 4).

**Fig. 5:**
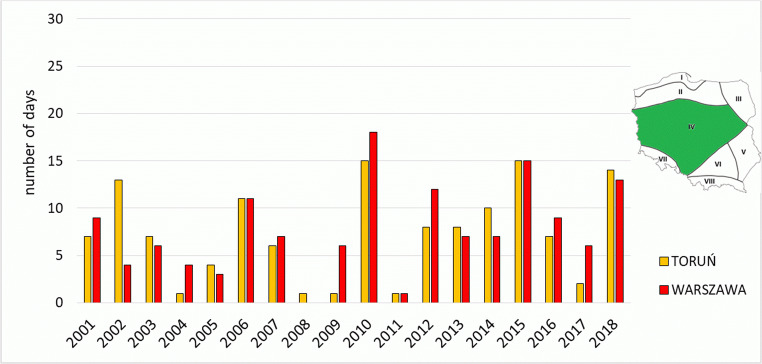
Number of days with heat stress categories (SHS + VSHS) on the meteorological stations in Central Region (IV)

The presented stations of the Central Region (IV) are characterised by biothermal conditions related to heat stress (*strong* and *very strong heat stress*), typical of the majority of Poland. Those two categories were observed in stations in Toruń and Warsaw for 7 and 8 days in a season, respectively. The highest number of such days was recorded in 2010 and 2015 (in Warsaw 18 and 15 days, respectively, and in Toruń 15 days in each of the aforementioned years). It is related mainly to heat waves observed in those years in Poland. In the first half of the analysed multi-annual period, the number of such days was lower than in its second half—this concerns all the analysed stations in the region (Fig. 5, Table 3).

**Table 3:** Number of days with strong and very strong heat stress (UTCI > 32 °C)

REG.	STATION	2001–2009 [no. of days]	2010–2018 (no. of days)	Increase (no. of days)
		a	b	b-a
I	Świnoujście	29	28	− 1
	Łeba	6	17	11
II	Szczecin	40	53	13
	Chojnice	20	31	11
III	Białystok	54	69	15
	Suwałki	27	60	33
IV	Toruń	51	80	29
	Warszawa	50	88	38
	Wrocław	72	98	26
	Zielona Góra	49	73	24
V	Lublin-Radawiec	69	100	31
	Terespol	61	99	38
VI	Kraków-Balice	90	118	28
	Rzeszów-Jasionka	66	127	61
VII	Jelenia Góra	36	49	13
	Śnieżka	0	0	0
VIII	Zakopane	9	23	14
	Kasprowy Wierch	0	0	0

**Fig. 6:**
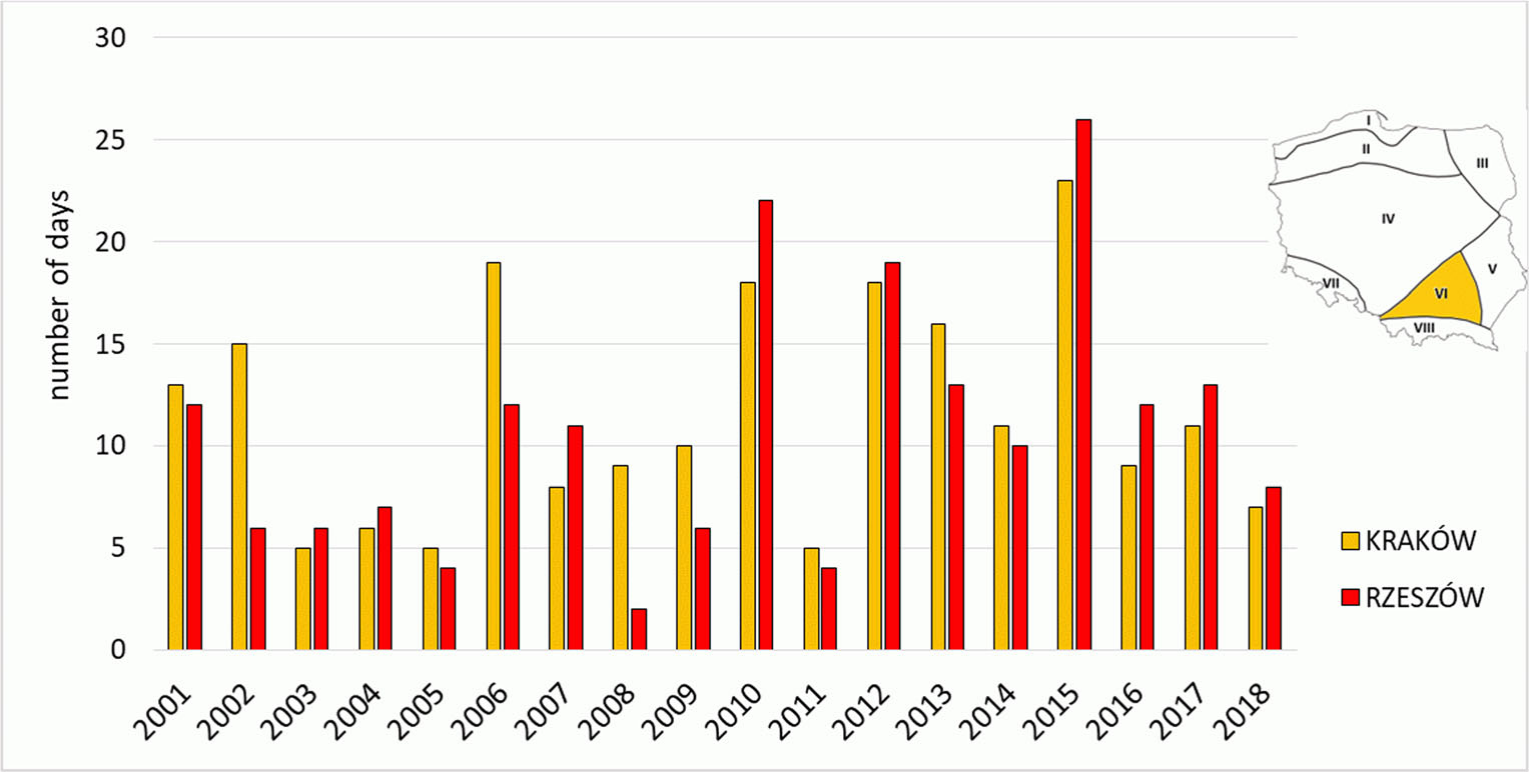
Number of days with heat stress categories (SHS + VSHS + EHS) on the meteorological stations in Upland Region (VI)

The Upland Region is the warmest region of Poland, which translates into the highest values of frequency of occurrence of *strong* and *very strong heat stress* in summer. The highest number of such days was recorded in 2015: 23 cases in Kraków and 26 cases in Rzeszów, due to a strong heatwave in the first half of August (Fig. 6). An increase in the number of days with *strong* and *very strong heat stress* in the second half of the analysed period is also evident. In Kraków in the years 2001–2009, 90 such days were recorded, and in the years 2010–2018—already 118. In Rzeszów, the number of such cases was 66 and 127, respectively (Table 3). In the Upland Region at 12.00 UTC, the highest UTCI value was recorded. It occurred in the station in Kraków on 08 August 2013 (40.8 °C), and was the highest value of the index determined for the noon term of observation in the analysed stations in the twenty-first century.

**Fig. 7:**
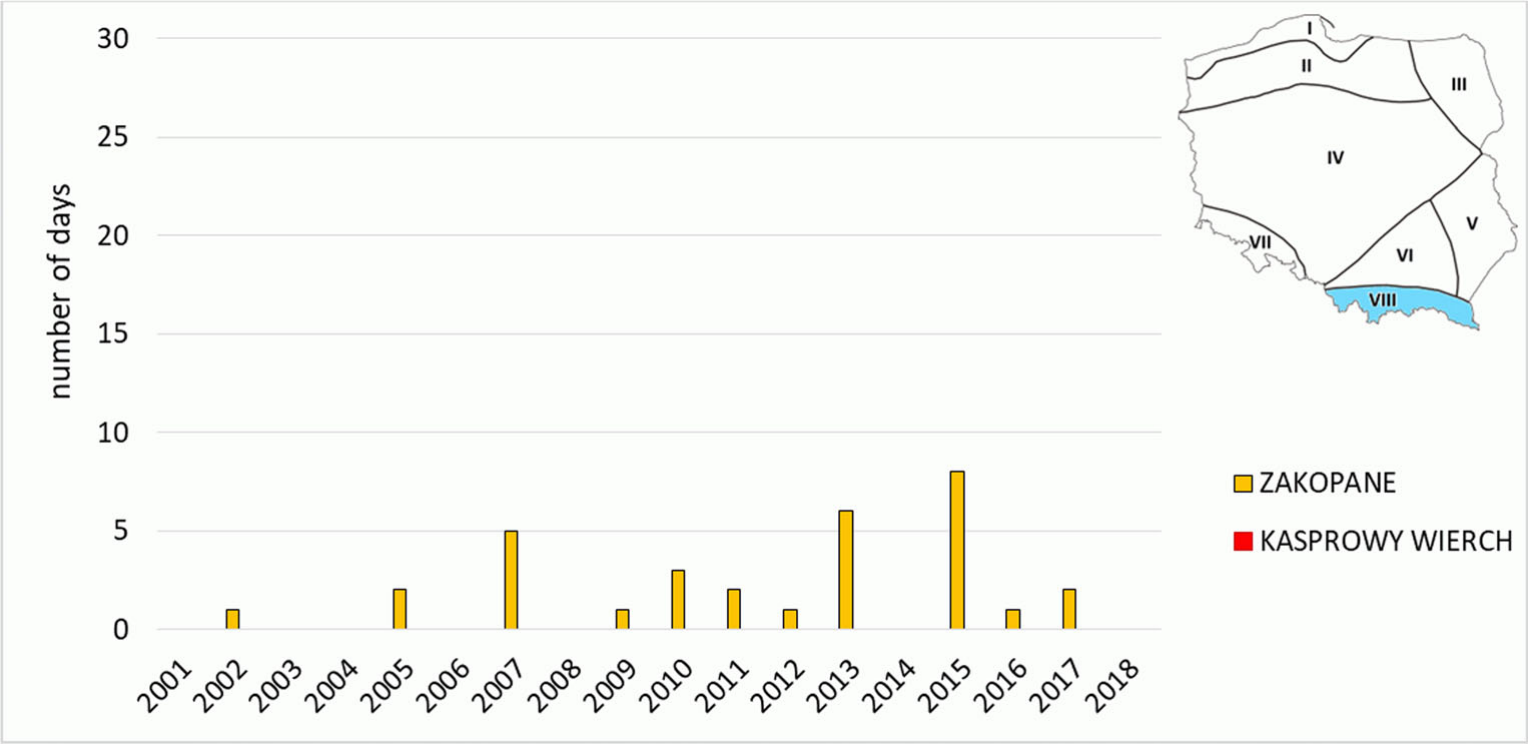
Number of days with heat stress categories (SHS + VSHS) on the meteorological stations in Carpathian Region (VIII)

In mountain areas, *strong heat stress* was observed only in stations located in mountain valleys. In Zakopane, they did not occur every year, and their highest number (8 cases) was observed in 2015 (Fig. 7). In Jelenia Góra, their number varied from 1 case in 2004 to 11 cases in 2015. In high mountain stations (Kasprowy Wierch and Śnieżka), no days with *strong heat stress* were recorded.

### *Categories with cold stress* (ECS + VSCS + SCS + MCS + SlCS, UTCI ≤ 9.0 *°*C)

**Fig. 8:**
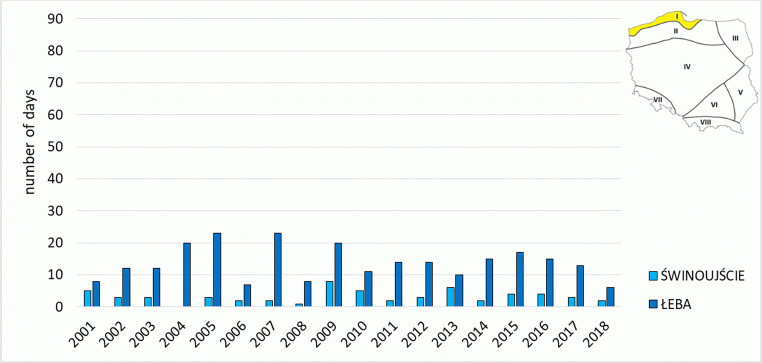
Number of days with cold stress categories (SCS + MCS + SlCS) on the meteorological stations in Coastal Region (I)

Regions located in the northern part of the country (Costal, Lakeland, and North-Eastern Region) are characterised by a higher number of days with classes related to cold stress, undesirable in regions associated with water recreation. The highest number of such cases was recorded in Łeba (from 6 cases in 2018 to 23 cases in 2005 and 2007) (Fig. 8). Heat stress in the region (particularly in the station in Łeba) is largely determined by air temperature lower than in the remaining stations of the Polish Lowland, and higher wind speed.

**Fig. 9:**
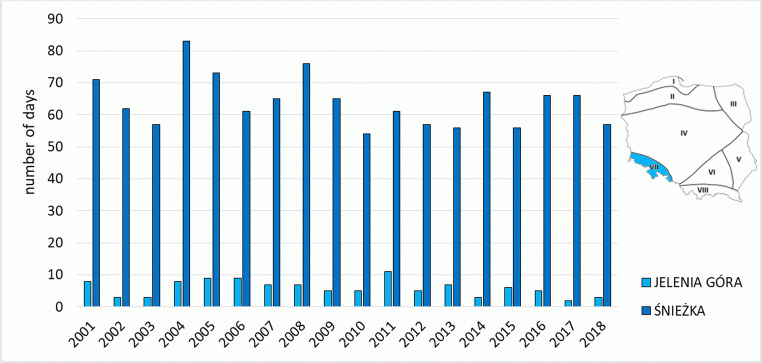
Number of days with cold stress categories (ECS + VSCS + SCS + MCS + SlCS) on the meteorological stations in Sudetic Region (VII)

Mountain regions, as the coldest regions in Poland, were characterised by a very high number of days with cold stress categories. On Śnieżka, from 54 to 83 of such days were recorded (Fig. 9), and on Kasprowy Wierch from 48 to 72 days with cold stress categories occurred. An average of 60 days with UTCI ≤ 9.0 *°*C was observed on Kasprowy Wierch, and 64 on Śnieżka. Although the meteorological station on Śnieżka is located approximately 400 m lower than the station on Kasprowy Wierch, higher wind speed values on Śnieżka translate into higher frequency of classes related to cold stress.

### Temporal analysis of the number of days with strong and very strong heat stress (UTCI > 32 °C)

Due to only 18 years of observation taken into the analysis, we divided our data in two 9-year periods to compare the difference in number of days with *strong* and *very strong heat stress*. In the twenty-first century, an increase in the number of days with *strong* and *very strong heat stress* was observed in almost all of the analysed stations, except for Świnoujście where a slight decrease in the number of such days occurred. In the first half of the analysed multi-annual period (2001–2009), from 6 cases in Łeba to 90 cases in Kraków were recorded. In the second half of the multi-annual period (2010–2018), from 17 such days in Łeba to 127 days in Rzeszów were observed. In high mountain stations, no conditions of *strong* and *very strong heat stress* occurred. Mean increase in all stations in comparison with years 2001–2009 and 2010–2018 was 21.3 days. It was the highest in the stations of the Central, Upland and South-East Region, which are the warmest regions in Poland. The highest increase by 61 days was observed in Rzeszów (Table 3).

Results of analysis of longer time period (1966–2015) and more stations (40) from Poland in half of stations show statistically significant increasing trend in the number of days with *strong* and *very strong heat stress*; the fastest increase is on the stations of south-western Poland and it is about 2 days per decade annually, and about 3 days per decade in summer months (Tomczyk and Owczarek 2020).

### Temporal analysis of UTCI in summer months

In summer, from June to August, in all analysed station the most frequently occurring class was *thermoneutral zone* (TZ, Fig. 2). In summer, the highest frequency of TZ was in June in coastal station Świnoujście (81.1%) and in near-mountain station Zakopane (81.3%). They were most frequent in June and the least frequent in July (Tables 4 and 5). Second class with the highest frequency was *moderate heat stress* (MHS), with the highest frequency in July in Warsaw (33.2%).

Biometeorological conditions with significant heat load for human body are characterised as *strong heat stress* (SHS), which appeared with minimum in June and maximum in July (Tables 4, 5 and 6). Kraków and Rzeszów are the cities with the highest frequency of *strong heat stress*. This is due to the location of these places in the vast valleys with a milder microclimate than the surrounding areas. The strongest recorded heat stress from *very strong heat stress* class (VSHS) occurred with the frequency below 2%, and it appears most often in August (Table 4, 5 and 6). The least frequent heat stress conditions (outside of mountainous areas) occurred in the Coastal Region, which confirms the cooling effect of the water reservoir in the warm period of the year. In the analysed stations, there were no conditions of the strongest UTCI class—*extreme heat stress* (EHS).

**Table 4:** The frequency of occurrence (%) UTCI classes at 12.00 UTC in June in Poland (2001–2018).

VI	ECS	VSCS	SCS	MCS	SlCL	TZ	MHS	SHS	VSHS	EHS	SUM
Świnoujście	-	-	-	1.1	6.3	81.1	10.2	1.3	-	-	100.0
Łeba	-	-	0.2	2.2	23.3	69.5	4.6	0.2	-	-	100.0
Szczecin	-	-	-	0.7	11.1	71.3	14.3	2.6	-	-	100.0
Chojnice	-	-	-	0.7	12.4	73.4	12.4	1.1	-	-	100.0
Suwałki	-	-	-	1.1	12.4	69.8	15.0	1.7	-	-	100.0
Białystok	-	-	-	0.2	5.7	71.9	19.6	2.6	-	-	100.0
Toruń	-	-	-	-	3.0	74.0	19.3	3.7	-	-	100.0
Warszawa	-	-	-	0.6	7.8	66.2	23.0	2.4	-	-	100.0
Zielona Góra	-	-	-	0.2	8.0	70.7	17.6	3.5	-	-	100.0
Wrocław	-	-	-	-	8.1	65.7	20.7	5.2	0.2	-	100.0
Terespol	-	-	-	-	3.3	73.2	19.6	3.9	-	-	100.0
Lublin	-	-	-	0.2	7.6	65.8	22.0	4.4	-	-	100.0
Kraków	-	-	-	1.1	8.7	62.4	20.9	6.7	0.2	-	100.0
Rzeszów	-	-	-	0.4	8.0	63.3	23.5	4.8	-	-	100.0
Zakopane	-	-	-	-	5.2	81.3	12.4	1.1	-	-	100.0
Kasprowy Wierch	-	0.7	9.8	28.5	35.7	25.2	-	-	-	-	100.0
Jelenia Góra	-	-	-	1.1	9.4	72.3	14.4	2.8	-	-	100.0
Śnieżka	0.7	6.5	18.7	25.2	26.3	22.6	-	-	-	-	100.0
Total	0.0	0.4	1.6	3.5	11.2	65.5	15.0	2.7	0.0	-	100.0

**Table 5:** The frequency of occurrence (%) UTCI classes at 12.00 UTC in July in Poland (2001–2018)

VII	ECS	VSCS	SCS	MCS	SlCL	TZ	MHS	SHS	VSHS	EHS	SUM
Świnoujście	-	-	-	-	2.2	68.9	23.7	4.8	0.4	-	100.0
Łeba	-	-	-	0.9	9.7	76.1	11.1	2.2	-	-	100.0
Szczecin	-	-	-	-	3.6	59.8	28.7	7.5	0.4	-	100.0
Chojnice	-	-	-	0.5	4.8	66.7	22.8	5.2	-	-	100.0
Suwałki	-	-	-	-	5.2	58.8	27.6	8.2	0.2	-	100.0
Białystok	-	-	-	-	0.9	54.8	33.0	11.3	-	-	100.0
Toruń	-	-	-	-	1.6	57.6	29.9	10.2	0.7	-	100.0
Warszawa	-	-	-	-	2.0	52.2	33.2	12.4	0.2	-	100.0
Zielona Góra	-	-	-	-	2.5	60.5	26.0	10.8	0.2	-	100.0
Wrocław	-	-	-	0.2	2.5	57.0	26.7	12.9	0.7	-	100.0
Terespol	-	-	-	-	2.2	51.9	32.3	13.4	0.2	-	100.0
Lublin	-	-	-	-	2.3	54.5	29.0	14.0	0.2	-	100.0
Kraków	-	-	-	-	3.8	52.5	28.3	13.8	1.6	-	100.0
Rzeszów	-	-	-	-	4.8	51.1	27.6	15.2	1.3	-	100.0
Zakopane	-	-	-	-	2.0	72.8	22.9	2.3	-	-	100.0
Kasprowy Wierch	-	0.7	3.0	22.6	35.5	38.2	-	-	-	-	100.0
Jelenia Góra	-	-	-	-	5.2	66.7	22.4	5.7	-	-	100.0
Śnieżka	-	5.4	15.6	22.9	23.8	32.3	-	-	-	-	100.0
Total	-	0.3	1.0	2.6	6.4	57.4	23.6	8.3	0.3	-	100.0

**Table 6:** The frequency of occurrence [%] UTCI classes at 12.00 UTC in August in Poland (2001–2018)

VII	ECS	VSCS	SCS	MCS	SlCL	TZ	MHS	SHS	VSHS	EHS	SUM
Świnoujście	-	-	-	-	1.1	71.4	23.7	3.6	0.2	-	100.0
Łeba	-	-	-	0.9	8.1	79.2	10.0	1.8	-	-	100.0
Szczecin	-	-	-	-	1.6	71.0	21.1	6.1	0.2	-	100.0
Chojnice	-	-	-	0.2	3.6	73.4	19.9	2.9	-	-	100.0
Suwałki	-	-	-	0.5	3.4	66.5	24.0	5.6	-	-	100.0
Białystok	-	-	-	-	1.6	60.5	29.7	8.2	-	-	100.0
Toruń	-	-	-	-	1.1	61.9	28.0	8.6	0.4	-	100.0
Warszawa	-	-	-	-	2.5	58.2	29.4	9.5	0.4	-	100.0
Zielona Góra	-	-	-	-	2.3	64.0	26.2	7.0	0.5	-	100.0
Wrocław	-	-	-	-	2.3	63.0	23.1	10.2	1.4	-	100.0
Terespol	-	-	-	-	1.8	57.5	29.4	10.8	0.5	-	100.0
Lublin	-	-	-	0.2	2.9	57.0	28.1	10.9	0.9	-	100.0
Kraków	-	-	-	0.4	2.3	54.5	27.6	14.3	0.9	-	100.0
Rzeszów	-	-	-	0.2	2.5	55.0	28.9	12.0	1.4	-	100.0
Zakopane	-	-	-	-	1.6	69.9	26.2	2.3	-	-	100.0
Kasprowy Wierch	-	0.2	3.8	17.4	36.4	42.2	-	-	-	-	100.0
Jelenia Góra	-	-	-	0.2	3.4	70.2	19.3	6.5	0.4	-	100.0
Śnieżka	-	3.8	9.3	25.8	25.1	35.8	0.2	-	-	-	100.0
Total	-	0.2	0.7	2.5	5.8	61.7	21.9	6.7	0.4	-	100.0

In the summer season, *very strong* and *extreme cold stress* (VSCS and ECS) occurred rarely and were recorded only at mountain stations (Śnieżka 5.4% and Kasprowy Wierch 0.3%). VSCS was most often recorded in June and what is interesting, in the warmest month of the season—July rather than in August. ECS appeared only in June on Śnieżka. The occurrence of such cold stress conditions is caused by the location of these stations at high altitude above sea level (above 1600 m), which affects the decrease in air temperature and the increase in wind speed.

Slightly smaller cold stress, i.e. *moderate cold stress* (MSC) and *strong cold stress* (SCS), except for the mountain stations mentioned above (Kasprowy Wierch with 28.3% and Śnieżka with 39.1% total frequency), also occurred relatively often in Coastal Region, in Łeba (most often in June 2.4%). This is probably the effect of high wind speeds from the Baltic Sea and lower air temperature, which clearly cools the human body also during the summer.

### Temporal analysis of UTCI > 32 °C in summer days

Days with *strong* and *very strong heat stress* are occurring most often in July. At particular stations, this frequency was from about 2.2% in Coastal Region (Łeba) to 16.5% days in Upland Region (Rzeszów). Less often such days were observed in August from 1.8 in Coastal Region (Łeba) to 15.5% in Upland Region (Kraków). In June, those frequencies were from 0.2 in Coastal Region to 6.9 in Upland Region (Kraków).

However, detailed analysis of average daily frequency of SHS + VSHS days showed both temporal and spatial variations (Table 7). First, Coastal Region as well as both mountain regions (Sudetic and Carpathian) do not show clear periods of increase and decrease in number of such days. The only exception is Jelenia Góra, where at the end of July and in the beginning of August such days occurred in 3–5 years in an 18-year analysed period (with the frequency between 3 and 5 days per 18 years, which corresponds to 15–30% during that time). In Lakeland Region, this period of elevated frequency lasted from 26th July to 3rd August (up to 30%, which corresponds with up to 5 days during 18 years.). In remaining regions (North-Eastern, South-Eastern, Upland and Central) such days occurred more often, and they appeared during four distinguishable periods with increased frequency. The first period, with the highest frequency of occurrence from 3 to 7 days per 18 years (15–40%), lasted from 26th of July to 13th of August. The second one lasted from 14th to 22nd of July (with frequency from 2 to 6 days per 18 years, which is 10–33% frequency). The third and fourth were 3–7 July and 15–20 August; however, the latter was not observed in North-Eastern Region.

**Table 7:**
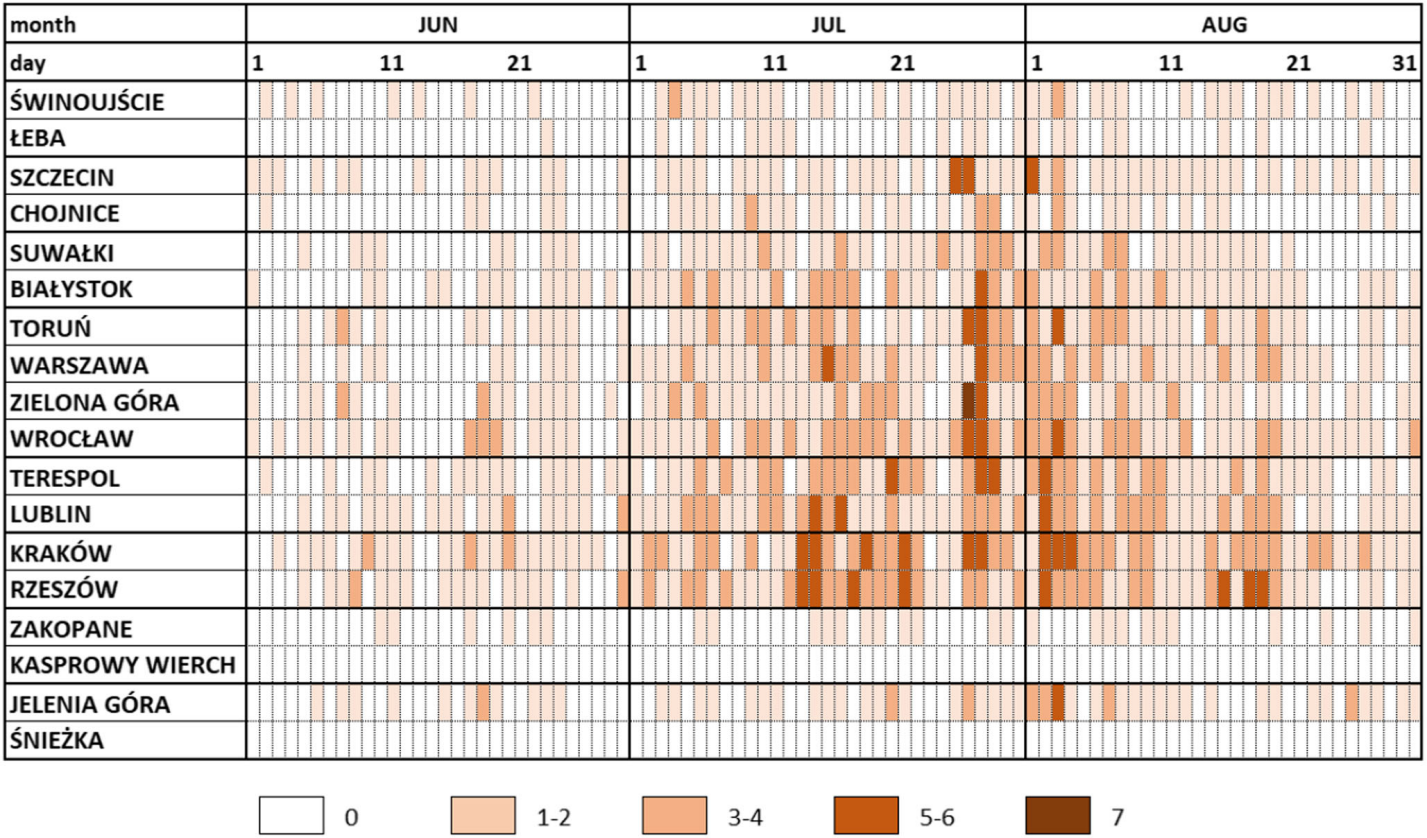
The number of days with *strong* and *very strong heat stress* in Poland (2001–2018).

Such situation is not very favourable for beach tourism at the Baltic Coast and in Lakeland Region, because even in tropical beaches, tourists prefer hotter weather (Rutty and Scott 2015), and in Poland, those two regions do not have particular periods with favourable (*strong* and *very strong heats stress*) conditions. In Central, Upland and South- and North-Eastern Regions, such conditions have appeared quite often. In those regions, there are many historical cities, and heat stress conditions can limit activities connected with urban tourism in those places (Scott and Lemieux 2010).

### Heat wave in August 2015

In the first half of August 2015, a very strong heat wave occurred over Eastern Europe, in terms of intensity and severity comparable with the heat wave from August 2003 over Western Europe (Hoy et al. 2016; Muthers et al. 2017; Krzyżewska and Dyer 2018). In this region, it was one of the longest and most severe heat waves in the twenty-first century (Hoy et al. 2016; Urban et al. 2017; Výberči et al. 2017). The wave was caused by the inflow of hot tropical air from the south and an extensive area of high pressure (Hoy et al. 2016; Bartoszek and Krzyżewska 2017). In Poland, the highest total number of cases (from 24 h observations) with UTCI > 32 °C during the heat wave occurred in the Central Region in Opole: 104 cases (hourly terms) with conditions of *strong* and *very strong heat stress*. Two hottest days of the heatwave in Poland occurred on 7–8 August when thermal records were established in many stations (Krzyżewska et al. 2019).

**Fig. 10:**
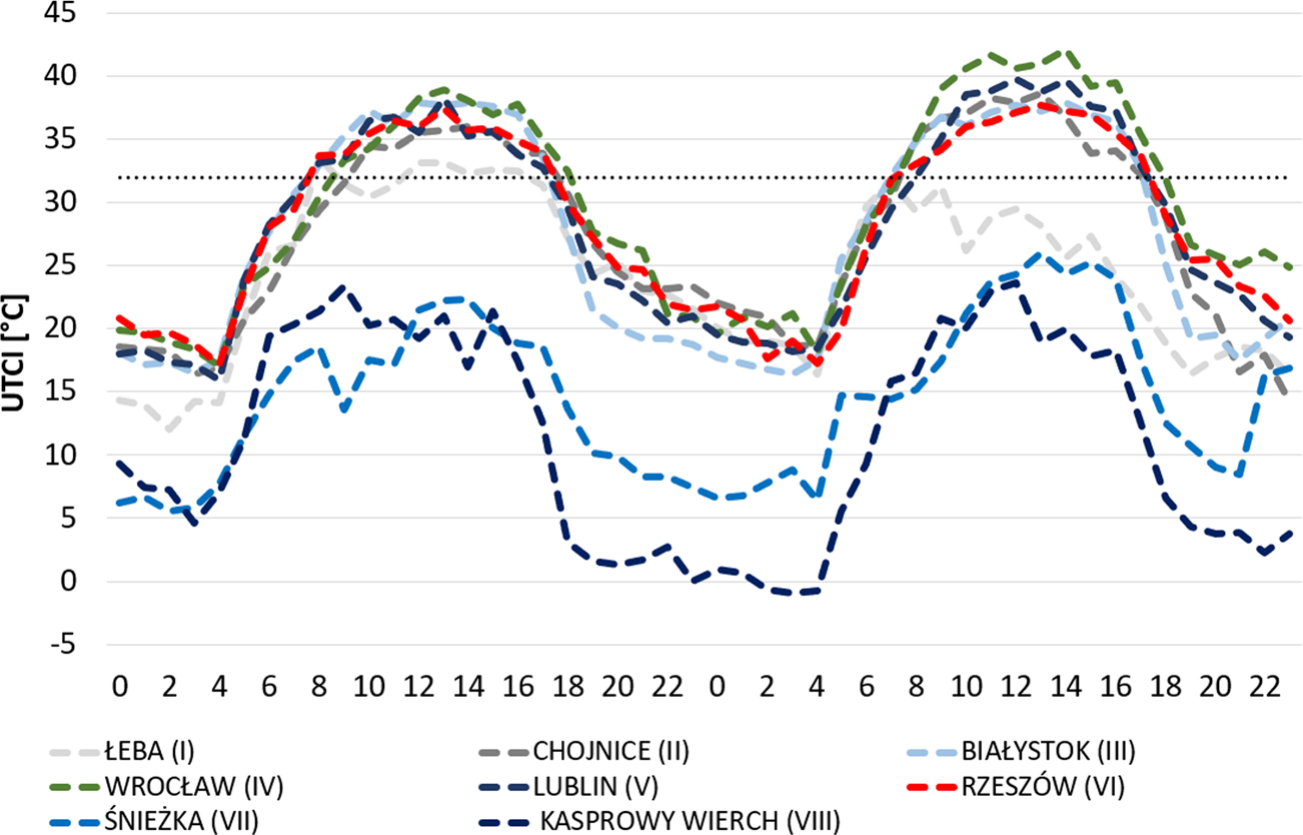
UTCI during two hottest days (7–8 August) of the 2015 heatwave in Poland. Horizontal dotted black line is the UTCI > 32 °C

During those two hottest days, conditions of *strong* and *very strong heat stress* were recorded (Fig. 10). Such conditions were maintained from approximately 8 to 19 UTC in the majority of the analysed stations (with the exception of some stations located in the north of the country at the seaside, e.g. Łeba, and in the south and the mountains, e.g. Śnieżka and Kasprowy Wierch, where no such conditions occurred). The highest UTCI value (42.1 °C) was recorded in Wrocław on 8 August 2015 at 14 UTC. There, on that day, conditions of *very strong heat stress* were maintained the longest, i.e. for 8 hours.

## Discussion and conclusions

Summer is the season with the highest intensity of tourist traffic. Therefore, it is very important to determine biometeorological conditions, particularly in popular tourist destinations such as coastal and mountain areas, especially in the times of climate changes.

In Poland in summer, *thermoneutral* conditions are prevalent (56–75% of summer days), as confirmed by research of other authors (Błażejczyk and Kunert 2011; Nidzgorska-Lencewicz and Mąkosza 2013; Kuchcik 2017; Kolendowicz et al. 2018). The second most frequently occurring class is *moderate heat stress* (18 to 29%). In coastal and mountain areas, however, the values are lower, and *cold stress* occurs more frequently. Only in Łeba (Coastal Region) and on mountain stations (Kasprowy Wierch, Śnieżka) UTCI values in summer were lower than − 13 °C (SCS + VSCS + ECS), with 1 case of *strong cold stress* in Łeba, 91 cases of *strong cold stress* and 9 cases of *very strong cold stress* in Kasprowy Wierch and 240 cases of *strong cold stress*, 86 cases of *very strong cold stress* and 4 cases on *extreme cold stress* in Śnieżka. Łeba stands out in those terms, due to its location in the middle coast, open to the cooling effect of the Baltic waters, resulting in the strongest bioclimatic stimuli in the region (Chabior and Korpalska-Chabior 2006). Research conducted for a longer measurement series showed that the number of days with cold stress rapidly decreases (Kolendowicz et al. 2018), which in the case of tourism in the coastal areas is a positive phenomenon (Rutty and Scott 2015). In mountain areas, high variability of biothermal conditions is observed, determined by the land relief and absolute height. Both in the Sudetic and Carpathian Regions, stations located in high mountain areas, there are more days with cold stress. Stations located in mountain valleys show higher frequency of thermal comfort. This is confirmed by research by Miszuk et al. (2016). According to the authors, in the summer half-year in Jelenia Góra, days with *thermoneutral* conditions are prevalent (in June their frequency reaches up to 60–75% of days in a month), and on Śnieżka they occur much more seldom (in August their frequency is 40% of days in a month). Based on measurements conducted on the Hala Gąsienicowa, Błażejczyk and Kunert (2010) point out that in the Carpathians (Tatra Mountains), in summer, conditions with *thermoneutral zone* and *slight cold stress* should be particularly expected*,* and *moderate cold stress* and *moderate heat stress* occur sporadically*.* Therefore, in mountain areas in the summer half-year, conditions favourable for practicing among others walking tourism occur, because even in the case of intensive physical activity, the human organism is not subject to excessive thermal stress related to high temperatures.

The Central Region is among areas characterised by milder stimuli of thermal environment (Błażejczyk and Kunert 2011). This part of Poland, belonging to the European Lowland, is characterised by small height differences, facilitating the flow of air masses from over the Atlantic Ocean. In summer months, higher air temperature values and lower wind speeds are recorded here more than in other areas of the country (Woś 1999; Kuchcik et al. 2013), affecting the development of local biothermal conditions. In the region, during the heat wave in 2015, the highest UTCI value was recorded (42.1 °C), which occurred on 8 August at 14 UTC in Wrocław.

The Upland Region is among the warmest bioclimatic regions in Poland. The number of days with *strong* and *very strong heat stress* in the stations of the region was the highest. In Rzeszów, 193 such cases were recorded in the twenty-first century (which gives on average 10.7 day per summer season). In Kraków, their number reached 208 (which is 11.5 day per summer season). Moreover, in Kraków, the highest UTCI value at 12.00 UTC was recorded on 08 August 2013 (40.8 °C).

As mentioned previously, UTCI is the index which corresponds well with air temperature and other meteorological elements influencing human heat balance. This is confirmed by spatial variations of biothermal conditions in summer season in Poland. Most cases of *strong* and *very strong heat stress* are observed in Central, Upland and South-Easter Region (Kuchcik et al. 2013), when in summer months, there is higher air temperature, lower wind speed and more days with cloudiness < 20% (Woś 1999). In Coastal, Sudetic and Carpathian Region, where *strong* and *very strong heat stress* class occurs rarely (Kuchcik et al. 2013), air temperature in summer season is lower and wind speed is higher. In summer, fewer ‘sunny or with little clouds amount’ days are observed at the Baltic Coast in the north and in mountain areas in the south (Woś 1999).

On the other hand, this relationship between UTCI and other meteorological elements is visible in observed and forecast increase in number of days with *strong* and *very strong heat stress*, with the increase of air temperature and sunshine radiation and no changes in wind speed (Błażejczyk et al. 2015).

The relatively high number of days with *strong* and *very strong heat stress* is a limitation for urban tourism (Scott and Lemieux 2010). That can be a problem for cities from Central and Upland Regions, which are popular tourist destinations in Poland. For example, in 2019 Kraków was visited by 14.5 million tourists (according to research of Tourism Organization of Małopolska, published by www.krakow.pl). Besides Kraków, there are multiple objects of UNESCO Heritage Sites, for example Old Towns of Warszawa and Toruń, Wieliczka and Bochnia Royal Salt Mines, Auschwitz Birkenau—German Nazi Concentration and Extermination Camp, Krzemionki Prehistoric Striped Flint Mining Region or Centennial Hall in Wrocław (Więckowski 2006). Also in those regions, there are many sanctuaries, churches and objects of religious tourism—for example Wadowice (birth place of pope John Paul II), Sanctuary of the Mercy Mother of God in Częstochowa (Więckowski 2006).

Another aspect of increasing number of days with *strong* and *very strong heat stress* is increased mortality, especially during heat waves (Muthers et al. 2017; Kuchcik 2017; Robine et al. 2008; Urban et al. 2017; Výberči et al. 2017). In twenty-first century in Poland, the strongest heatwave (mega-heatwave) occurred in first half of August in 2015 with the highest number of UTCI > 32 °C cases (from 24 hours observations) in Central and Upland Regions. Those areas in Poland are very highly populated (Gawryszewski 2006) and have relatively low (in comparison with the rest of the country) shares of green areas, like forests, meadows and pastures (Degórska 2006). One of the results can be decreased soil moisture, which contributes to longer and more severe summer heatwaves (Fischer et al. 2007). Unfortunately, during the past heatwave events in Poland, those areas were particularly affected (Krzyżewska and Dyer 2019).

Days with *strong* and *very strong heat stress* did not appear with the same frequency during all three summer months. The month with the highest frequency is July, followed by August. Such days are least frequent in June. Based on the data from 2001 to 2018, there are 4 potential periods of occurrence of such days, with two most intense being 26.07–13.08 and 14–22.07.

An increase in the number of days with *strong* and *very strong heat stress* was observed in the years 2010–2018 in comparison with the years 2001–2009. This corresponds with the increase in the number of days strenuous for the human organism in terms of heat stress in the twenty-first century (Di Napoli et al. 2018), including heatwaves, forecasted by IPCC (2014). In Poland, it particularly concerns the Central, Upland and South-East Region. Such conditions may have a negative effect on the intensity of tourist traffic in cities such as: Kraków, Toruń, Warsaw or Wrocław, but may also improve conditions favouring sea- and sunbathing in the Coastal Region (I).
